# Light induced transthylakoidal proton gradient is a key signal driving the downward migration of motile diatoms in sediments

**DOI:** 10.1038/s41598-025-07737-5

**Published:** 2025-07-01

**Authors:** Jérôme Morelle, Johann Lavaud, Douglas A. Campbell, Silja Frankenbach, João Serôdio

**Affiliations:** 1 CESAM – Centre for Environmental and Marine Studies and Department of Biology, University of Aveiro, Campus de Santiago, 3810-193 Aveiro, Portugal; 2 LEMAR - Laboratory of Marine Environmental Sciences, UMR 6539 CNRS, Univ Brest, Ifremer, IRD, Institut Universitaire Européen de la Mer, Technopôle Brest-Iroise, Plouzané, France; 3 Biology Department, Mount Allison University, Sackville, NB Canada

**Keywords:** Light-induced migration, Photosynthetic stress, Photoprotection, Chlorophyll fluorescence, Non-photochemical quenching (NPQ), Quantum yield, Microbial ecology, Light responses, Microbial ecology

## Abstract

Pennate diatoms are photosynthetic microorganisms capable of directed motility in response to light. In sedimentary habitats, many epipelic pennate diatoms exhibit photophobic migration under high light, a behaviour critical for avoiding photodamage and key to ecological success. While the ecophysiological significance of this behaviour is well-documented, the mechanisms linking light sensing to motility remain poorly understood. This study investigated whether the transthylakoidal proton gradient (ΔpH), generated under high light, intervenes in the signal transduction mechanism driving photophobic migration. The impact of the ΔpH inhibitors Nigericin and Carbonyl cyanide 4-(trifluoromethoxy)phenylhydrazone (FCCP) on the vertical migration of benthic pennate diatoms was monitored using non-destructive imaging chlorophyll fluorometry on intertidal diatom-dominated microphytobenthos biofilms. The results showed that ΔpH inhibition significantly reduced the downward, high light-avoiding, migratory response, supporting the hypothesis that ΔpH plays a central role in mediating this response. Additionally, results showed that the effective quantum yield of PSII and non-photochemical quenching (NPQ) were impacted by ΔpH inhibition with a dose-dependent effect. These findings strongly support ΔpH as an integrative signal linking physiological and behavioural photoprotection mechanisms and suggest that ΔpH may also modulate intracellular signalling, explaining the efficient capacity of pennate diatoms to cope with high light exposure in benthic habitats.

## Introduction

Diatoms are ubiquitous unicellular photosynthetic organisms found in virtually every aquatic habitat, where they play crucial roles in ecosystem functioning. As primary producers, diatoms are responsible for approximately 40% of total carbon fixation in marine ecosystems and contribute to about 20–25% of the combined carbon fixation in all aquatic and terrestrial ecosystems^1,2^. In addition to their pivotal role in carbon sequestration supporting aquatic trophic networks, diatoms also contribute extensively to nutrient cycling, oxygen production, and sediment stabilization in benthic habitats^3^.

Among the diatoms inhabiting benthic habitats, the raphid pennate group has notably thrived in colonizing intertidal areas. Species of this group dominate microbial communities known as microphytobenthos (MPB), forming dense and highly productive biofilms that cover extensive intertidal sedimentary areas^4^. Uniquely among diatom groups, the epipelic pennates exhibit directed motility, hypothesized to be a major factor explaining their evolutionary success, diversification, and productivity in sedimentary habitats. This motility is thought to enable these species to cope with and actively exploit the resource heterogeneity of the sedimentary habitat, namely by using vertical migration to respond to changes in light intensity incident at the sediment surface^5–11^.

In addition to migratory movements synchronized with daylight and tide, epipelic benthic diatoms perform phototaxis, resulting in an upward vertical migration to the sediment surface under moderate irradiance and a downward vertical migration under irradiance below and above this range. Epipelic diatoms display photophobic behaviour under high light conditions, optimizing photosynthesis while minimizing photoinhibitory stress and cellular photodamage^8,10,12–20^. This response, which varies with species and physiological state, is well-documented in the literature. However, while the ecophysiological significance of this behaviour is well established, the underlying mechanisms triggering the vertical migration responses to light intensity are still largely unexplored, and little is known about how variations in light intensity are transduced from sensing to cellular movement.

Light sensing by motile diatoms has primarily been studied in terms of the cellular location of photosensitive regions^21,22^, responses to the duration and spectrum of light stimuli^13^ and the putative photoreceptors involved^23,24^. Sensing of light can rely upon photoreceptors that activate specific signalling cascades to mediate the response to light^25^. However, in photosynthetic cells such as diatoms, light is also absorbed by photosynthetic pigments capturing a much larger fraction of the light spectrum than dedicated regulatory photoreceptors, activating mechanisms that can also regulate responses to light via retrograde pathways^26^.

One such transduction pathway is linked to the build-up of the light induced transthylakoidal proton gradient (ΔpH). When light energy is captured by photosynthetic pigments; electrons are transferred through the electron transport chain located in the thylakoid membrane. This electron flow drives two key processes that contribute to the formation of ΔpH, (i) the protons (H⁺ ions) released into the thylakoid lumen originating from the splitting of water molecules, and (ii) the protons pumped from the stroma into the lumen by the cytochrome b_6f._ complex^27^. Together, these processes generate the proton gradient and ΔpH essential for ATP synthesis^28^. The build-up of ΔpH is also involved in photophysiological processes such as the activation of the xanthophyll cycle and non-photochemical quenching (NPQ) processes^29^. These key roles of ΔpH in the regulation of physiological light responses lead to the hypothesis that it could also serve as a signal transduction mechanism mediating the induction of photophobic downward vertical migration of epipelic diatoms.

The main objective of this study was thus to test the hypothesis that the sensing and transduction response to high light in epipelic pennate diatoms are mediated by the transthylakoidal proton gradient (ΔpH) generated under high light conditions. To test this hypothesis, experiments were carried out on natural sediment samples colonized by diatom-dominated MPB biofilms. ΔpH inhibitors were applied on samples in conjunction with high light exposure and vertical migration was quantified non-destructively by measuring changes in surface microalgal biomass using imaging Pulse Amplitude Modulation (PAM) fluorometry. Under the hypothesis, it was expected that the MPB biofilms treated with ΔpH inhibitors would exhibit a reduced downward migration in response to high light as compared to untreated counterparts.

## Results

### Taxonomic composition

The cell suspension obtained from the sediment used in the Nigericin experiment was largely dominated by pennate diatoms, with minor contributions from cyanobacteria and Euglenoids (Table 1). Among the pennate diatoms, the majority were small species (< 30 µm), primarily belonging to the genus *Navicula* spp. A smaller proportion of the assemblage consisted of larger cells, mainly of the genera *Gyrosigma* sp., *Cylindrotheca* sp., and *Entomoneis* sp., with a few individuals from *Nitzschia* sp., *Cocconeis* sp., and *Surirella* sp.

**Table 1:** Relative abundance of microalgae taxa present in the samples used in the Nigericin and FCCP experiments. The percentage is based on the count of cells of a given species relative to the total number of cells counted.

Species	Nigericin experiment (%)	FCCP experiment (%)
Diatoms		
Small pennate diatom (< 30 µm), mainly *Navicula* spp.	91.4 ± 2.9	89.3 ± 2.6
*Gyrosigma* sp.	2.2 ± 1.1	1.4 ± 0.9
*Cylindrotheca* sp.	1.7 ± 1.3	1 ± 0.7
*Entomoneis* sp.	1.3 ± 1.9	0.7 ± 0.7
*Nitzschia* sp.	0.3 ± 0.5	2.2 ± 1.2
*Pleurosigma* sp.	0	1.5 ± 0.8
*Cocconeis* sp.	0.3 ± 0.4	0.8 ± 0.6
*Diploneis* sp.	0	0.8 ± 0.7
*Surirella* sp.	0.5 ± 0.6	0
Unidentified pennate diatom (> 30 µm)	0	1 ± 0.8
Others		
Cyanobacteria	1.2 ± 0.6	0.4 ± 0.3
Euglenoids	1.1 ± 1	0.9 ± 0.7

In the FCCP experiment, the cell suspension was similarly dominated by pennate diatoms, with a small presence of cyanobacteria and Euglenoids (Table 1). The small-sized diatoms, mostly from the genus *Navicula* spp., were again the dominant group. The larger cells included representatives from *Nitzschia* sp., *Pleurosigma* sp., *Gyrosigma* sp., *Cylindrotheca* sp., and individuals from *Entomoneis* sp., *Cocconeis* sp., and *Diploneis* sp.

### Effect of ΔpH inhibitors on surface biomass

**Fig. 1:**
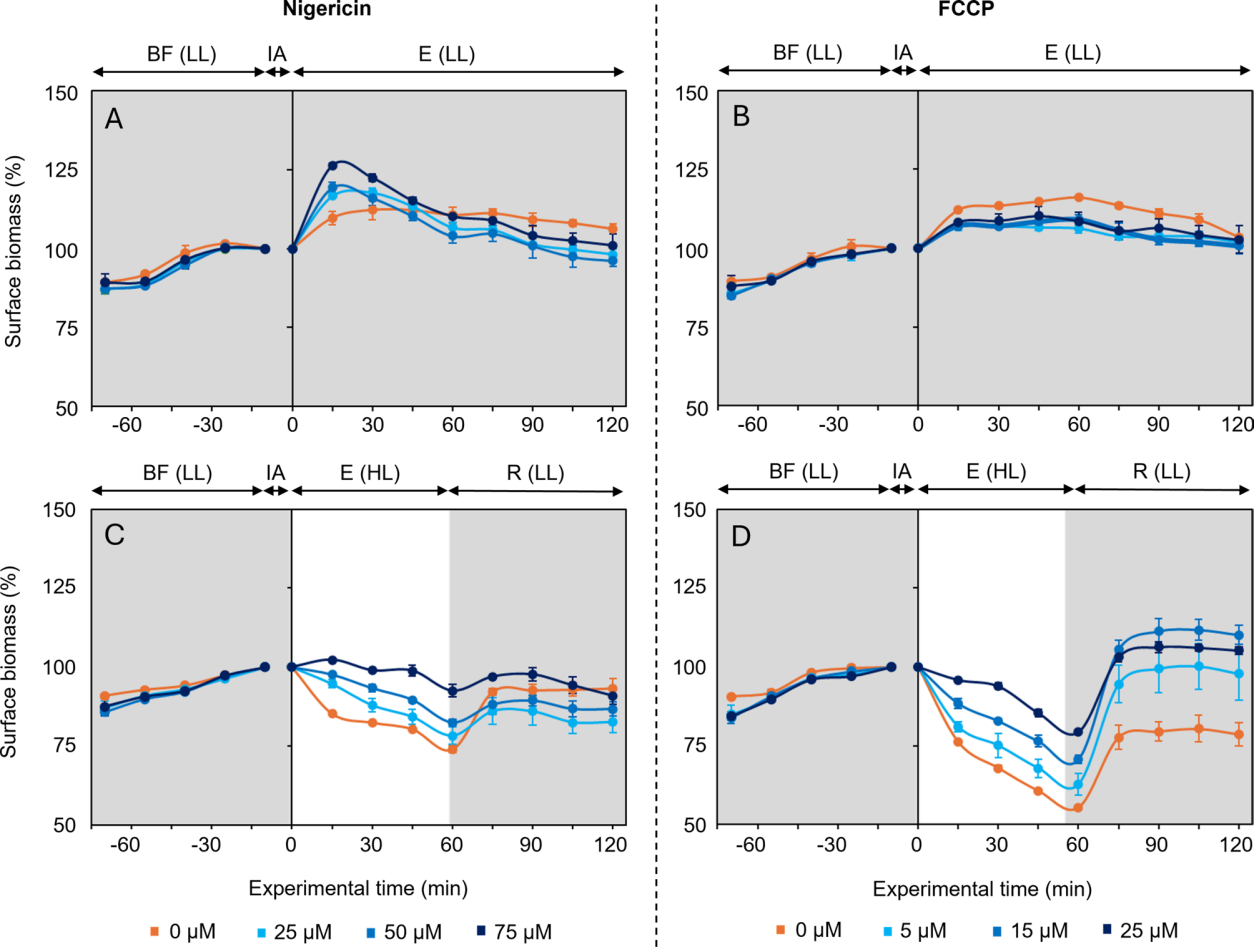
Response of sediment surface biomass proxy (F_s_) to light treatments. (mean ± standard error; n = 3). Light treatments consisted of 120 min under low light exposure (E(LL) – **A**,**B**) or 60 min under high light exposure (E(HL)), followed by 60 min of recovery under low light (R(LL) –**C**,**D**). During biofilm formation (BF), values are expressed as relative percentages of those obtained at the end of this period, set at 100%. After the inhibitor addition (IA: Nigericin – **A**,**C** or FCCP – **B**,**D**), values are expressed as relative percentages of those measured immediately after IA.

Under low light conditions and prior to the introduction of the ΔpH inhibitors, all replicates exhibited a vertical upward migration of cells. This was shown by a significant increase in F_s_ values during 45 min (*p* < 0.01, Fig. 1). During the subsequent time of low light exposure before introduction of the ΔpH inhibitors, the rate of migration slowed down, and the surface biomass stabilized, with no significant variation in F_s_ values. The stabilization of F_s_ values was considered indicative of the full formation of the biofilm at the sediment surface, marking the time for the application of the inhibitors, just before 0 min (Fig. 1).

Under steady low light conditions, in the absence of Nigericin (0 µM) F_s_ increased slightly over 15 min (Fig. 1A), with little variation during the rest of the exposure period. Addition of Nigericin amplified the initial increase in F_s_ values within the first 15 min, in a dose-dependent manner. After 15 min, F_s_ values gradually declined, and by 60 min, no significant differences were observed between any Nigericin concentrations and the control. Similarly, with FCCP, F_s_ values increased slightly during the first 15 min and remained stable throughout the rest of the experiment (Fig. 1B), with no significant differences found among concentrations. These results indicate that the inhibitors alone had no major effect on vertical migration under low light.

Following a switch to high light conditions, the control samples with no added inhibitor (0 µM) exhibited a significant decrease in F_s_ values. In the Nigericin experiment (Fig. 1C), the control showed a significant decrease in surface biomass (p < 0.05) of 26.1 ± 1.3% between 0 to 60 min of high light exposure, reflecting downward photophobic migration. Addition of 25, 50 or 75 µM nigericin progressively inhibited this downward migration (Fig. 1C).

Similarly, in the FCCP experiment (Fig. 1D), the control also exhibited a significant decrease in F_s_ (p < 0.001) of 44.7 ± 0.8% during the 60 min of high light exposure, again showing downward photophobic migration. Addition of 5, 15, 25 µM FCCP showed progressive, partial, inhibition of this downward migration (Fig. 1D).

### Effects of ΔpH inhibitors on ΔF/F_m_'

**Fig. 2:**
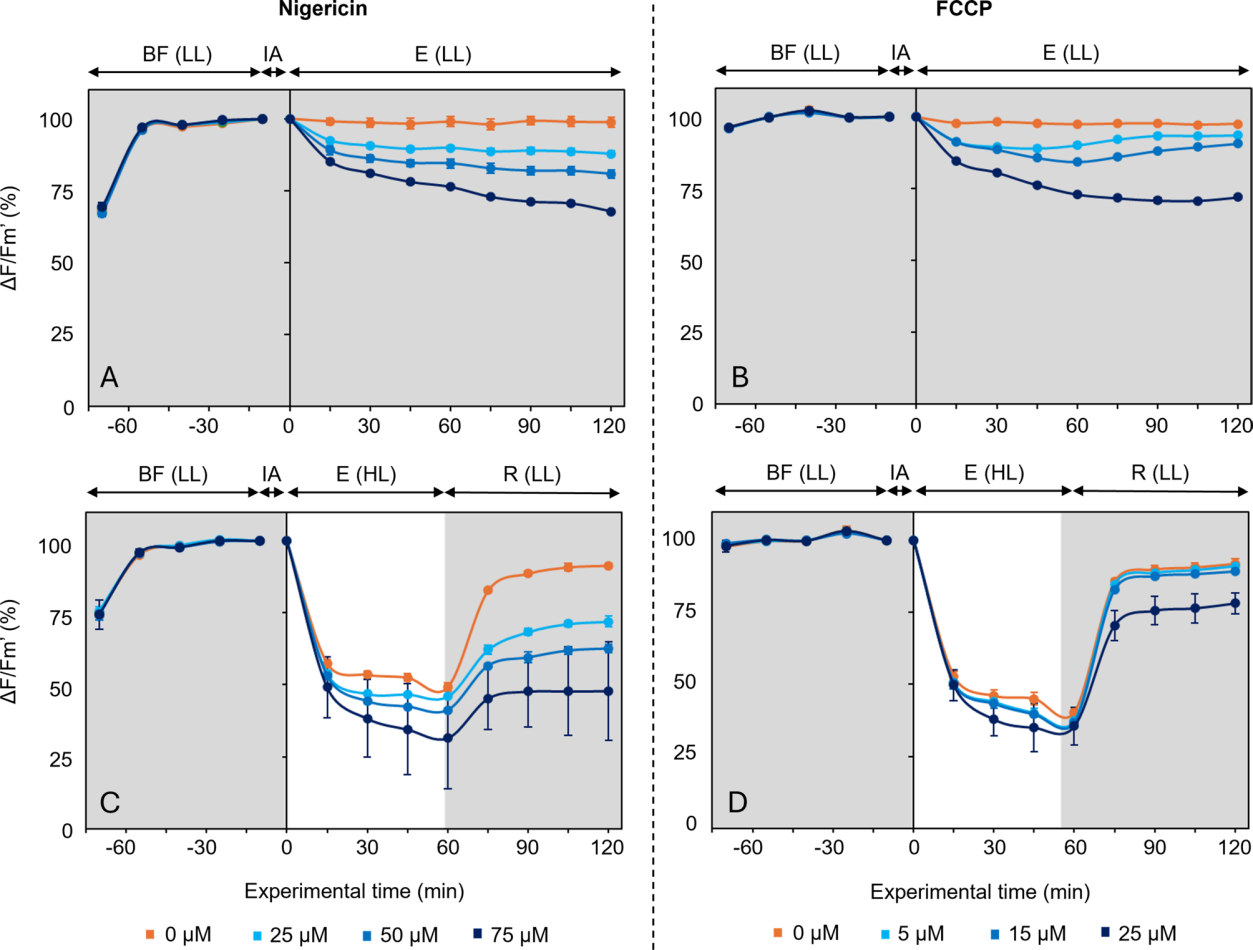
Response of the effective quantum yield of PSII (ΔF/F_m_ʹ) to light treatments (mean ± standard error; n = 3). Light treatments consisted of 120 min under low light exposure (E(LL) – **A**,**B**) or 60 min under high light exposure (E(HL)), followed by 60 min of recovery under low light (R(LL) – **C**,**D**). During biofilm formation (BF), values are expressed as relative percentages of those obtained at the end of this period, set at 100%. After the inhibitor addition (IA: Nigericin – **A**,**C** or FCCP – **B**,**D**), values are expressed as relative percentages of those measured immediately after IA.

Prior to the introduction of the ΔpH inhibitors, ΔF/F_m_' values increased significantly during 45 min (p < 0.001; Fig. 2), reaching 0.64 and 0.65 for each experiment. No significant variation was observed during the following 15 min.

After the application of the inhibitors, samples kept under low light showed a dose-dependent effects on ΔF/F_m_' (Fig. 2A,B). For Nigericin (Fig. 2A), samples started with ΔF/F_m_' values of 0.64, not significantly different across wells. While the controls did not vary significantly during the exposure, Nigericin-treated samples exhibited a significant dose-dependent decrease (p < 0.05), with each concentration causing significantly different effects from each other (p < 0.001). From 60 to 120 min under low light, no significant changes in ΔF/F_m_' were observed for the control and for the lowest Nigericin concentration (25 µM), reaching final values of 0.63 and 0.57, respectively. However, the higher concentrations (50 and 75 µM) induced small but significant decreases in ΔF/F_m_', with values dropping to 0.51 and 0.44 at the end of the experiment. With FCCP (Fig. 2B), a significant decrease (p < 0.05) in ΔF/F_m_' was also observed after application of the inhibitor. Although no significant differences were found between 5 µM and 15 µM during the first 30 min, all concentrations showed a significant dose-dependent decrease (p < 0.001) after this time. From 60 to 120 min, no significant changes in ΔF/F_m_' were observed for the control and the highest FCCP concentration (25 µM), with final values of 0.64 and 0.50, respectively. In contrast, intermediate concentrations (5 and 15 µM) resulted in significant increases in ΔF/F_m_' values, that rose to 0.63 at 5 µM and 0.62 at 15 µM.

Under high light exposure, the variation in ΔF/F_m_' after the addition of Nigericin showed that all samples started with similar values, and no significant differences between the different concentrations were observed at subsequent time points. A significant decrease was observed (Fig. 2C) with values dropping from an average of 0.63 to 0.29 after 30 min (p < 0.001). After this point, ΔF/F_m_' values remained stable until the end of the high light exposure (p > 0.1). When the light was switched from high to low, from 60 to 120 min, the control recovered to ΔF/F_m_' averaging 0.57, while treated samples recovered to lower values, reaching only 0.45, 0.39, and 0.31 for 25, 50, and 75 µM, respectively.

With the addition of FCCP (Fig. 2D), ΔF/F_m_' values were also not significantly different between concentrations at the start of the exposure, averaging 0.67, and remained similar at each subsequent time point. A significant decrease was observed after 15 min of high light exposure, with values dropping to 0.25 at the end of the exposure (p < 0.05). When the light intensity was switched back to low, from 60 to 120 min, ΔF/F_m_' reached a mean value of 0.59 with no significant differences between treatments. However, in relative terms, the highest FCCP concentration resulted in a lower proportion of ΔF/F_m_' recovery (Fig. 2D).

### Effects of ΔpH inhibitors on Y(NPQ)

**Fig. 3:**
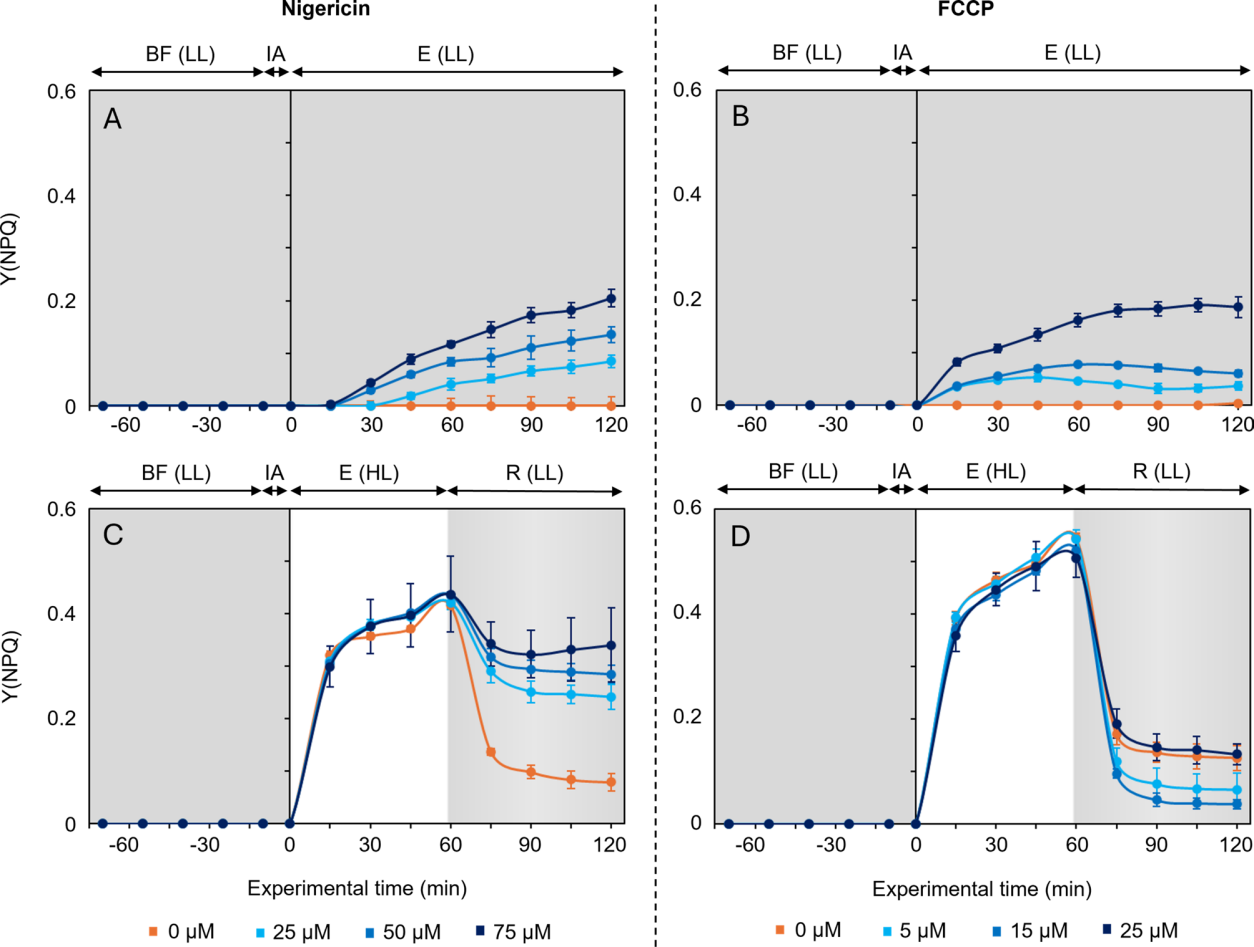
Response of the non-photochemical quenching index Y(NPQ) to light treatments. (mean ± standard error; n = 3). During biofilm formation (BF) and inhibitor addition (IA: Nigericin – **A**,**C** or FCCP – **B**,**D**), light treatments consisted of 120 min under low light exposure (E(LL) –**A**,**B**) or 60 min under high light exposure (E(HL)), followed by 60 min of recovery under low light (R(LL) – **C**,**D**).

After application of Nigericin, the Y(NPQ) values measured under low light showed different variations depending on the concentration applied (Fig. 3A). The control showed no significant variation during the 120 min of low light exposure. In contrast, treated samples showed a significant increase (*p* < 0.001) with a dose dependent effect. The values increased up to 0.08 ± 0.02, 0.14 ± 0.02, and 0.21 ± 0.03, respectively with 25, 50, and 75 µM Nigericin.

The same pattern was observed in the samples kept under low light and treated with FCCP (Fig. 3B). In contrast with the control, the values of the other concentrations were significantly higher (*p* < 0.001) with a dose dependent effect. During the 120 min of low light exposure, no significant changes were noted in the control or for the lowest concentrations (5 and 15 µM), with average values of 0.04 ± 0.01, and 0.07 ± 0.01, respectively. At the highest FCCP concentration (25 µM), Y(NPQ) values increased significantly up to 0.19 ± 0.03 (Fig. 3B).

When exposed to high light and treated with Nigericin (Fig. 3C), the Y(NPQ) values didn’t show significant differences between the concentrations applied, this for each time point. Therefore, all values increased significantly over time, with values from the different time point being significantly different from each other (*p* < 0.001), up to an average value of 0.43 ± 0.01 after 60 min of exposure. When the light intensity was switched to low, Y(NPQ) decreased to 0.08 ± 0.02 in the controls, while dropping to 0.24 ± 0.03, 0.28 ± 0.02, and 0.34 ± 0.09 with 25, 50, and 75 µM Nigericin, respectively.

Under high light, the Y(NPQ) values measured for the different FCCP concentrations (Fig. 3D) also showed no significant differences for each time point. All values showed a significant increase (*p* < 0.001) up to an average value of 0.53 ± 0.04 at the end of the exposure. When the light was switched to low, the Y(NPQ) values decreased across all concentrations, with final values showing no significant differences between them, averaging 0.09 ± 0.04.

## Discussion

The objective of this study was to test the hypothesis that in epipelic diatoms the sensing and transduction response to high light is mediated by the high light-induced transthylakoidal proton gradient (ΔpH) formed in the plastids. Our results support this hypothesis, by demonstrating that, under high light exposure, diatom-dominated biofilms treated with ΔpH inhibitors exhibit a dose-dependent decrease in photophobic vertical migration compared to untreated biofilms.

The taxonomic composition (Table 1) and the initial upward migration (Fig. 1) observed under low light conditions and prior to inhibitor addition confirms that the sediment samples used in this study were effectively dominated by pennate diatoms capable of responding to irradiance changes through vertical migration. Furthermore, the ΔF/F_m_' values (Fig. 2), exceeding 0.6 across all samples, indicates a good photophysiological state of the cells. Together, they indicate that the sampled biofilms were reliable models for assessing the impacts of ΔpH inhibitors (Nigericin and FCCP) on vertical migration under varying light conditions.

The results showed that the two inhibitors significantly inhibited the high light-induced downward migration following a dose-dependent pattern. The observation that the migratory response of treated cells was significantly smaller than the one of untreated samples, and that this difference increased with the ΔpH inhibitor concentration, is a strong indication that these chemicals disrupted the ability of the cells to respond behaviourally to high light.

Even though ΔpH plays a crucial role in ATP production, which is necessary for migration, it is unlikely that the observed slowdown in migration was due to an ATP shortage. First, the energy required for vertical migration is minimal, estimated at only 0.12 pJ for a benthic diatom to glide over 400 µm^30^. Furthermore, the upward migration observed before inhibitors addition confirms that the cells already had sufficient energy to initiate and sustain this movement. Additionally, during this hour where cells were exposed to low light, they likely synthesized and stored energy-rich molecules such as carbohydrates or lipids. These stored molecules can be metabolized to regenerate ATP when conditions do not support direct ATP production, allowing diatoms to sustain metabolic activity even under suboptimal conditions^31^. Therefore, the reduction in migration observed under high light intensity and in the presence of ΔpH inhibitors is more likely due to disruptions in cellular processes rather than a direct lack of ATP.

The results observed during high light exposure in control samples confirm that the diatom cells exhibited a strong photophobic migratory behaviour. The distinct vertical migration patterns observed in the control samples, with a deeper migration in the FCCP than in the Nigericin experiment, were likely caused by factors such as different endogenously controlled behaviour^32^. Additionally, variations in the light dose experienced by the communities in the days (or weeks) prior to sampling may have contributed to these differences, as previous light history can influence photophysiological acclimation and, consequently, migratory responses^33^. However, this aspect does not compromise the overall observation of the ΔpH inhibitor effect on the photophobic migration. This effect was confirmed by the observation that the downward migration was not induced under low light, even when treated with ΔpH inhibitor, reinforcing the idea that high light was the only driver of the observed downward migration.

Previous studies have demonstrated that benthic diatoms activate various cellular processes, photoprotective mechanisms, to mitigate potential damage under high light exposure. These processes include vertical migration to sediment layers with reduced light intensity^6,15,16^ and energy dissipation via non-photochemical quenching (NPQ)^34,35^. This NPQ plays a crucial role in dissipating excess light energy as heat, thereby preventing the overproduction of reactive oxygen species (ROS), which can harm proteins, pigments, and lipids, ultimately reducing photosynthetic efficiency.

In diatoms, NPQ consists of a high-energy state quenching (q_E_), usually reversible within 10–30 min, and a photoinhibitory quenching (q_I_), which requires longer recovery times due to the de novo synthesis and activation of photoinactivated PSII D1 proteins^35^. q_E_ is regulated by ∆pH, the xanthophyll cycle^35^, and light-harvesting complex (LHCX) proteins^36^. ∆pH activates pH-sensitive enzymes, promoting the conversion of diadinoxanthin (DD) to diatoxanthin (DT)^37,38^. DT accumulation being the primary driver of energy dissipation under high light^39–41^. Additionally, ∆pH generation via mitochondrial ATP hydrolysis contributes to DT accumulation even in darkness^42,43^. A sufficient ∆pH threshold is therefore necessary to ensure the full NPQ activation for an effective photoprotection under fluctuating light^44^.

In this study, the inhibited migration following ∆pH inhibition was anticipated to prolong cell exposure to high light, which under untreated conditions would typically trigger an increase in Y(NPQ) due to the need for enhanced energy dissipation. However, despite a confirmed inhibition of migration, no significant difference in Y(NPQ) levels under high light were observed for cells treated with ∆pH inhibitors. Nonetheless, under low light, a significant dose-dependent increase in Y(NPQ) occurred (Fig. 3A,B) despite no notable changes in F_s_. Likewise, during low light recovery after high light (Fig. 3C,D), Nigericin and FCCP showed concentration-dependent partial blockage of the relaxation of YNPQ induced under high light.

Under low light, the dose-dependent increase in Y(NPQ) suggests that ∆pH inhibitors enhanced energy dissipation even in low light conditions. Since changes in Y(NPQ) are typically associated with modifications in LHCX protein levels or the xanthophyll pool size^35^, which are unlikely to occur over short experimental durations, it is more likely that ∆pH inhibition disrupted proton-coupled transport or altered the protonation state of regulatory proteins involved in NPQ induction, resulting in prolonged or intensified quenching. In diatoms, energy dissipation under low light has been suggested to be driven by a tight regulation of both diadinoxanthin de-epoxidation and diatoxanthin epoxidation^35^, and by ∆pH generation via mitochondrial ATP hydrolysis, which contributes to DT accumulation even in darkness^42,43^. These mechanisms may have been altered under ∆pH inhibition, sustaining NPQ despite a weak proton gradient^35^. Since Y(NPQ) and ΔF/Fm’ are generally inversely correlated, the increased energy dissipation observed under low light likely contributed to the observed significant and dose-dependent decrease in ΔF/F_m_'. However, it is important to note that the lowest values reached remained relatively high, generally above 0.5, indicating that the microphytobenthic biofilms maintained a strong photophysiological state.

The inhibition of ∆pH most likely inhibited the diadinoxanthin de-epoxidation enzyme, limiting DD-to-DT conversion, and/or accelerating the diatoxanthin epoxidation enzyme^35^. However, the significant Y(NPQ) accumulation under high light, even in the presence of inhibitors, suggests that this conversion remained sufficient to sustain q_E_ up to saturation. Alternatively, an auxiliary short-term energy dissipation mechanism may have compensated for suppressed ∆pH-dependent quenching, maintaining overall dissipation levels. One possibility is an increase in q_I_, involving temporary PSII inactivation, which could have contributed to the observe sustained NPQ. This is supported by the results of the Nigericin experiment since, during recovery, Y(NPQ) values in the controls relaxed to significantly lower levels than in treated samples, suggesting the activation of slow recovery processes such as D1 protein replacement after PSII photoinactivation (q_I_). In contrast, the results of the FCCP experiment showed no differences between control and treated samples during the recovery, suggesting that q_I_ was not present. This is further supported by the ΔF/F_m_′ values, which recovered to higher levels in both control and FCCP-treated samples, indicating preserved PSII efficiency. In the Nigericin experiment, ΔF/F_m_′ remained significantly lower during recovery, consistent with the sustained Y(NPQ) and indicative of a more persistent PSII downregulation, possibly consistent with q_I_. This may be linked to a larger migratory capacity in FCCP-treated samples, as indicated by the change in F_s_ values (Fig. 1). Cells might have migrated sufficiently to prevent excessive light exposure, thus avoiding q_I_. Indeed, it has been suggested that q_I_ is mainly triggered when q_E_ is exceeded and that migration prevents cells from experiencing saturating light levels^45^. Nonetheless, q_I_ is generally triggered by longer exposures and higher light intensities than those used in this study^46^, suggesting that it may not be the only mechanism responsible for the sustained Y(NPQ) observed during recovery in Nigericin-treated samples. In addition to q_I_, other mechanisms previously discussed for low light conditions could also contribute to maintaining energy dissipation during the recovery period. These processes (mitochondrial ATP-driven ∆pH generation and/or xanthophyll cycle enzyme activities) may remain altered after light stress, especially in the presence of disrupted proton gradients, thereby sustaining Y(NPQ) levels even after the high light exposure.

Overall, these results highlight the complex interaction between ∆pH, the xanthophyll cycle, and alternative regulatory pathways contributing to NPQ modulation under varying light conditions. Also to be noted is that interpreting NPQ measured in migratory biofilms is challenging, as its calculation depends on F_m_ and F_m_' values, both simultaneously influenced by NPQ activation and by vertical migration. Although this study does not establish a direct causal link between NPQ and vertical migration, it supports the idea that both processes are closely linked via ∆pH regulation.

The relationship between migration and NPQ may involve signalling components beyond ΔpH, particularly Ca^2+^ signalling. ΔpH inhibitors like FCCP and Nigericin can affect not only the transthylakoidal ΔpH but also proton gradients across other membranes^42^, potentially influencing intracellular pH and membrane transport^47^, and especially Ca^2+^ storage and transport which play crucial roles in cellular function^48^.

In diatoms, Ca^2+^ was identified as a key secondary messenger, involved in responses to stresses^24,49–51^ and potentially in photophobic migration, cytoskeletal reorganization, and gliding motility^13,52–56^. The EukCatA ion channels, responsible for Na⁺ and Ca^2+^ transport, are voltage-dependent^57,58^ and thus tightly linked to proton gradients through the membrane potential. Ca^2+^ influx through EukCatA channels were shown to be essential for Ca^2+^-dependent gliding motility most likely in relation with processes associated with the secretion of polysaccharide or the regulation of cytoskeleton dynamics^57^. Therefore, variations of ΔpH might influences Ca^2+^ signalling and, consequently, cellular and migratory responses to light stress. This study did not investigate directly the effects of variations in intracellular Ca^2+^ concentrations, but it appears reasonable to suggest that the disruption of proton gradients by FCCP and Nigericin likely impaired Ca^2+^ transport systems. This disruption might lead to a breakdown in the cellular signalling pathways essential for photophobic migration by interfering with the secretion of adhesive polymers, cytoskeletal reorganization, and the activation of other photoprotective mechanisms, such as NPQ, as Ca^2+^ signalling has also been showed to be involved in regulating light-harvesting complex dynamics^59^.

Our findings emphasize the critical role of ΔpH in regulating photophobic migration in benthic diatoms. Inhibition of ΔpH by Nigericin and FCCP disrupts this migratory response, confirming that ΔpH is a key factor in the mechanism that enables diatoms to avoid excessive light exposure. The effects of ΔpH inhibition on NPQ and ΔF/F_m_' further underscore the central role of the transthylakoidal proton gradient in the photophysiological response of diatoms. This represents a case of indirect photosensing by the photosynthetic apparatus^60^, where physiological and behavioural photoprotection are closely linked and triggered by a common sensing mechanism. We propose that the impact of ΔpH inhibition may be related to alterations in intracellular signalling pathways, particularly Ca^2+^ signalling. Further research is needed to explore the potential role of Ca^2+^ signalling, offering deeper insights into the photoadaptive strategies of benthic diatoms in their natural environment.

## Methods

### Sampling

In August and September 2024, samples of sediment colonized by diatom-dominated microphytobenthos were collected during diurnal low tide in an intertidal mudflat of the Ria de Aveiro, Portugal (Vista Alegre; 40°35′00.9"N 8°41′11.5"W). Immediately after sampling, the sediment was homogenized and distributed into two separate 24-well cell culture plates (Orange Scientific, Braine-l′Alleud, Belgium). For each well plate, 12 wells were filled with 2.8 mL of homogenized sediment, measured using a 5 mL syringe (HENKE-JECT, Seoul, Republic of Korea). 200 µL of autoclaved seawater was added to each well to prevent desiccation, and the well plates were kept in the dark at room temperature until the beginning of the experiment, i.e., 1 h before the diurnal low tide of the following day.

### Taxonomic identification

The remaining sampled sediment was used to collect cells using the lens tissue technique (Eaton and Moss 1966). The resulting cell assemblages were resuspended in 100 mL of natural seawater, 2 mL was fixed using Lugol (5% v/v), and kept at 4 °C for future identification of the dominant species present. Identification was conducted under a Motic AE31 Inverted Microscope using 1 mL of the assemblage placed in a Sedgewick-Rafter chamber. Ten squares were analysed within the chamber. All cells were counted and identified to the genus level. The mean percentage of each identified taxon was calculated from the counts across the 10 squares (n = 10). This procedure was repeated for each assemblage.

### Biofilm formation

**Fig. 4:**
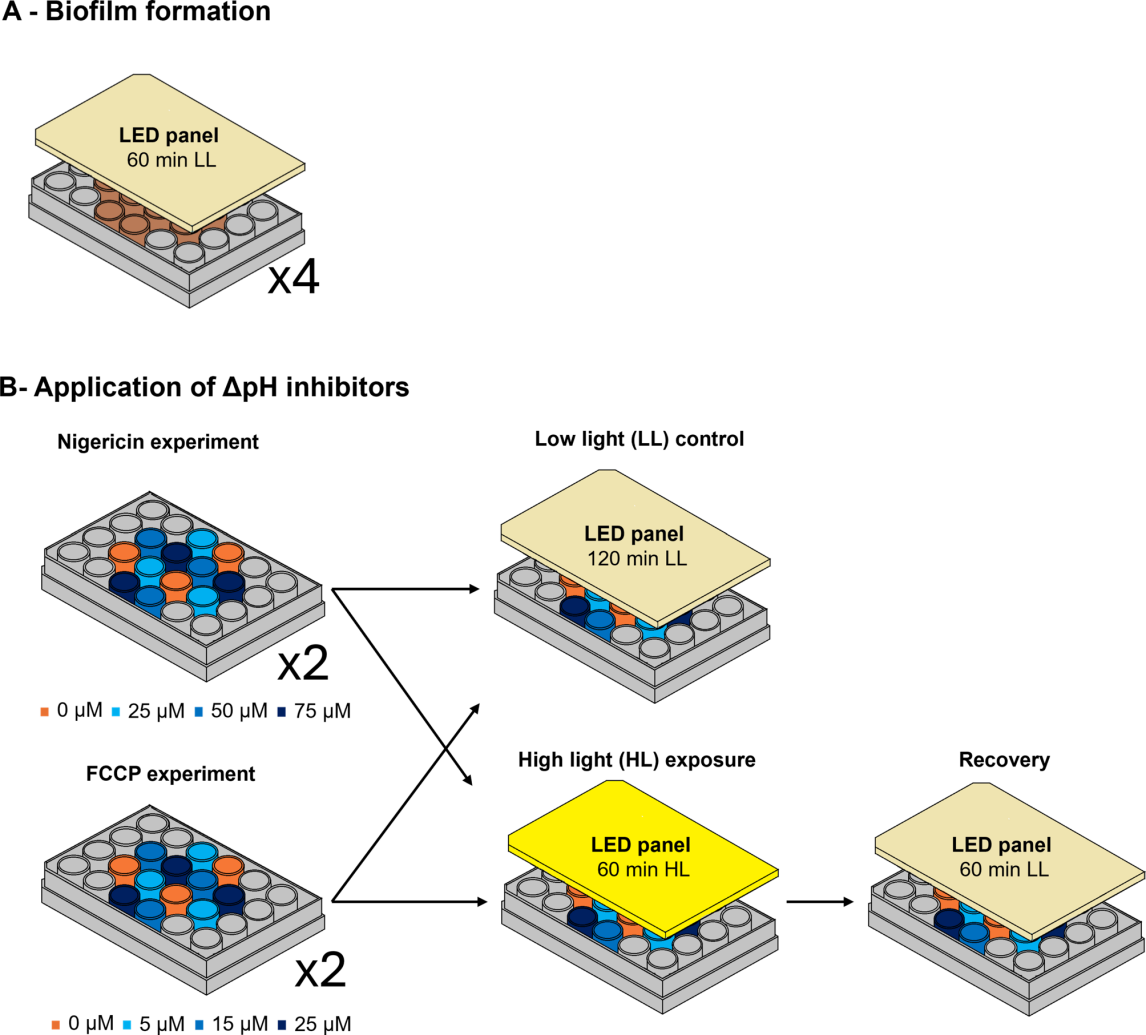
Experimental workflow. (**A**) Biofilm formation under low light (LL; 103 ± 5 µmol photons m⁻^2^ s⁻^1^) for 60 min under a LED panel and (**B**) application of ΔpH inhibitors Nigericin or FCCP, followed by distinct light treatments: 120 min of continuous low light as a control, and 60 min of high light (HL; 1000 ± 47 µmol photons m⁻^2^ s⁻^1^) to induce photophobic migration, followed by 60 min of low light to monitor photophysiological recovery and migration.

One hour before the start of the diurnal low tide of the day following sampling, each well plate was positioned under a RGBW LED illumination panel^61^, with peak emission wavelengths of 630 nm (red), 525 nm (green), 465 nm (blue), and 380–780 nm (white). The intensity of each LED was adjusted to ensure a homogeneous low light field across all wells, corresponding to a mean photosynthetic photon flux density (PPFD) of 103 ± 5 µmol photons m⁻^2^ s⁻^1^. The incident light field was characterized by measuring the PPFD using a calibrated sensor (Mini Quantum Sensor LS-C, Heinz Waltz, GmbH, Germany) positioned at the same distance from the light source as the sediment surface. Measurements were taken every 5 mm across the diameter of each well to assess the homogeneity of light received at the sediment surface. The well plates were kept under this homogeneous low light for one hour to induce the upward migration of the microphytobenthic cells and self-forming of the biofilm (Fig. 4A). The formation of the biofilm was followed by regular measurements of the chlorophyll fluorescence parameter F_s_ used as proxy for surface biomass^18,62^.

### Application of ΔpH inhibitors

The following ΔpH inhibitors, known to inhibit the build-up of the transthylakoidal ΔpH, through different mechanisms, were applied: (i) Nigericin Sodium Salt (Cayman Chemical, Ann Arbor, USA), a compound that disrupts the transthylakoidal ΔpH by allowing potassium ions to pass into the chloroplast stroma through the thylakoid membrane by exchanging with protons (H⁺) accumulated in the thylakoid lumen, thereby decreasing the ΔpH^63^; (ii) Carbonyl Cyanide-p-trifluoromethoxyphenylhydrazone (FCCP, TCI Europe N.V., Zwijndrecht, Belgium), a compound that allows H⁺ to move freely across the thylakoid membrane without passing through the ATP synthase^64^. This process equalizes the proton concentration between the lumen and the stroma, effectively collapsing the ΔpH. The choice of two ΔpH inhibitors acting through different mechanisms was meant to ensure that the observed effects are effectively due to ΔpH variation and not to other indirect effects of each individual ΔpH inhibitor.

Stock solutions of the two ΔpH inhibitors were prepared immediately before their application. For each stock solution, 10 mg of Nigericin or FCCP was initially dissolved in 1 mL of absolute ethanol and then diluted with 99 mL of autoclaved seawater to achieve final concentrations of 133.9 µM Nigericin and 393.4 µM FCCP, respectively. Seawater was used to mitigate any effect of absolute ethanol.

After the formation of the biofilm, 200 µL of ΔpH inhibitor solution was added to each well, at different dilutions to achieve different concentrations in the sediment samples. The experimental design allowed application of four different inhibitor concentrations per plate, including a control, with three replicates. The concentrations used were chosen according to the range of values used on microalgae cultures in the literature^42,65,66^, but increased to compensate for dilution in the sediment, resulting in a final set of 0, 5, 15, and 25 µM for FCCP, and a set of 0, 25, 50, and 75 µM for Nigericin. The different concentrations were assigned to specific wells to ensure a balanced distribution of replicates across the plate, avoiding clustering of replicates of the same group, and to guarantee that the three replicates of each concentration experienced identical light intensities to those of other concentrations (Fig. 4B).

### Light exposure

Following the addition of the ΔpH inhibitors, each well plate was positioned under a different light environment (Fig. 4B): one plate was exposed to high light conditions, corresponding to 1000 ± 47 µmol photons m⁻^2^ s⁻^1^. The exposure to this light level was previously confirmed to induce downward photophobic vertical migration, enabling the investigation of any role ΔpH might play in this process. The second plate was kept under low light conditions (103 ± 5 µmol photons m⁻^2^ s⁻^1^) as control to assess the effect of the ΔpH inhibitors on the measured parameters, without the effect of high light on vertical migration. This light intensity was also previously confirmed to ensure that it did not induce photophobic migration. The high light treatment was applied for 60 min, followed by a 60-min low light period (103 ± 5 µmol photons m⁻^2^ s⁻^1^) to monitor the photophysiological recovery of the cells after the high light stress. The low light treatment was exposed to low light conditions only, for a duration of 120 min (Fig. 4B).

### Chlorophyll fluorescence measurements

Throughout the entire timeline of the experiment (including biofilm formation, before and after the application of ΔpH inhibitor, light exposure, and recovery), surface MPB biomass was measured every 15 min using an imaging chlorophyll fluorometer (Open FluorCam FC 800-O/1010, PSI, Drásov, Czech Republic; see Serôdio et al.^67^), composed of two red LED panels (621 nm emission peak, 40 nm bandwidth; MLS13 × 13, PSI), providing the measuring non-actinic light (0.1 μmol m⁻^2^ s⁻^1^), and two red LED panels providing saturating pulses (7500 μmol m⁻^2^ s⁻^1^; SL3500, PSI). A user-defined protocol, with a total duration of 2 s, was employed to measure the fluorescence parameters. Briefly, without any dark adaptation, the well plates were individually excited by the non-actinic measuring light for 1.4 s to determine the steady state level of fluorescence (F_s_), after which a high-intensity saturation pulse was applied for 0.6 s to determine the relative level of maximum fluorescence (F_m_')^68^. These fluorescence parameters were used to calculate the effective quantum yield of PSII (ΔF/F_m_' = (F_m_' − F_s_)/F_m_')^69^.

F_s_ was used as a surface MPB biomass proxy to monitor changes in chlorophyll *a* in the photic zone of the sediment resulting from downward or upward migration^18,62^. An increase in F_s_ was thus considered to reflect upward migration of the microphytobenthic cells to the sediment surface, while a decrease in F_s_ was considered indicative of a downward migration of the cells into the subsurface layers of the sediment^16,18^.

Additionally, to estimate light-induced photoprotection through thermal dissipation of energy, the non-photochemical quenching (NPQ) index was calculated after ΔpH inhibitor addition and throughout the rest of the experiment based on the fluorescence values (Y(NPQ) = F_s_/F_m_' − F_s_/F_m_)^70^, using F_m_ recorded right after the application of ΔpH inhibitor.

### Data and statistical analysis

Statistical tests were performed using the software Sigma-Plot V12. The Shapiro–Wilk normality test was applied to the residuals of the model to ensure the assumption of normality was met, and the Bartlett test was used to assess homogeneity of variances in the datasets. When those tests were validated, the significance of the differences between concentrations at each time point was assessed through a one-way ANOVA followed by a Tukey HSD test. When normality and variance conditions were not met, a non-parametric Kruskal–Wallis followed by a pairwise test (Dunn’s method) was performed. For comparisons across the experimental timeline, a repeated measures ANOVA was used to assess differences over time within a single experimental condition. To account for variations in F_s_ values across replicates, treatments, and especially between different experiments, the F_s_ values measured after the onset of experimental light exposure were expressed as percentages relative to those recorded immediately following the addition of ΔpH inhibitors, which served as a reference.

## Data Availability

The datasets generated and analyzed during this study are available from the corresponding author upon reasonable request.

## References

[1] Field, C. B., Behrenfeld, M. J., Randerson, J. T. & Falkowski, P. Primary production of the biosphere: Integrating terrestrial and oceanic components. *Science* **80** (281), 237–240 (1998). 10.1126/science.281.5374.237 9657713

[2] Armbrust, E. V. The life of diatoms in the world’s oceans. *Nature* **459**, 185–192 (2009). 19444204 10.1038/nature08057

[3] B-Béres, V. et al. Ecosystem services provided by freshwater and marine diatoms. *Hydrobiologia* **850**, 2707–2733 (2023). 10.1007/s10750-022-04795-y PMC8795964 35106010

[4] Hope, J. A., Paterson, D. M. & Thrush, S. F. The role of microphytobenthos in soft-sediment ecological networks and their contribution to the delivery of multiple ecosystem services. *J. Ecol.* **108**, 815–830 (2020).

[5] Admiraal, W. The ecology of estuarine sediment-inhabiting diatoms. *Prog. Phycol. Res.* **3**, 269–322 (1984).

[6] Kromkamp, J., Barranguet, C. & Peene, J. Determination of microphytobenthos PSII quantum efficiency and photosynthetic activity by means of variable chlorophyll fluorescence. *Mar. Ecol. Prog. Ser.* **162**, 45–55 (1998).

[7] De Brouwer, J. F. C. & Stal, L. J. Short-term dynamics in microphytobenthos distribution and associated extracellular carbohydrates in surface sediments of an intertidal mudflat. *Mar. Ecol. Prog. Ser.* **218**, 33–44 (2001).

[8] Consalvey, M., Paterson, D. M. & Underwood, G. J. C. The ups and downs of life in a benthic biofilm: Migration of benthic diatoms. *Diatom Res.* **19**, 181–202 (2004).

[9] Nakov, T., Beaulieu, J. M. & Alverson, A. J. Accelerated diversification is related to life history and locomotion in a hyperdiverse lineage of microbial eukaryotes (Diatoms, Bacillariophyta). *New Phytol.* **219**, 462–473 (2018). 29624698 10.1111/nph.15137 PMC6099383

[10] Serôdio, J. Diatom motility: Mechanisms, control and adaptive value. *Diatom Gliding Motility*10.1002/9781119526483.ch7 (2021).

[11] Poulsen, N., Davutoglu, M. G. & Zackova Suchanova, J. Diatom adhesion and motility. In *The Molecular Life of Diatoms* (eds Poulsen, N. et al.) (Springer, 2022).

[12] Cohn, S. A. & Disparti, N. C. Environmental factors influemcing diatom cell motility. *J. Phycol.* **30**, 818–828 (1994).

[13] Cohn, S. A. et al. Characterisation of the diatom photophobic response to high irradiance. *Diatom Res.* **19**, 167–179 (2004).

[14] Underwood, G. J. C. et al. Patterns in microphytobenthic primary productivity: Species-specific variation in migratory rhythms and photosynthesis in mixed-species biofilms. *Limnol. Oceanogr.* **50**, 755–767 (2005).

[15] Serôdio, J., Coelho, H., Vieira, S. & Cruz, S. Microphytobenthos vertical migratory photoresponse as characterised by light-response curves of surface biomass. *Estuar. Coast. Shelf Sci.* **68**, 547–556 (2006).

[16] Perkins, R. G. et al. Vertical cell movement is a primary response of intertidal benthic biofilms to increasing light dose. *Mar. Ecol. Prog. Ser.* **416**, 93–103 (2010).

[17] Ezequiel, J., Laviale, M., Frankenbach, S., Cartaxana, P. & SerÔdio, J. Photoacclimation state determines the photobehaviour of motile microalgae: The case of a benthic diatom. *J. Exp. Mar. Bio. Ecol.* **468**, 11–20 (2015).

[18] Laviale, M., Frankenbach, S. & Serôdio, J. The importance of being fast: comparative kinetics of vertical migration and non-photochemical quenching of benthic diatoms under light stress. *Mar. Biol.* **163**, 1–12 (2016).

[19] Barnett, A., Méléder, V., Dupuy, C. & Lavaud, J. The Vertical Migratory Rhythm of Intertidal Microphytobenthos in Sediment Depends on the Light Photoperiod, Intensity, and Spectrum: Evidence for a Positive Effect of Blue Wavelengths. *Front. Mar. Sci.* **7**, 1–18 (2020).32802822

[20] Prins, A., Deleris, P., Hubas, C. & Jesus, B. Effect of light intensity and light quality on diatom behavioral and physiological photoprotection. *Front. Mar. Sci.* **7**, 1–17 (2020).32802822

[21] Cohn, S. A. Chapter 13 photo-stimulated effects on diatom motility. *Compr. Ser. Photosciences* **1**, 375–401 (2001).

[22] Cohn, S. A. et al. Comparative analysis of light-stimulated motility responses in three diatom species. *Diatom. Res.* **30**, 213–225 (2015).

[23] Jaubert, M., Bouly, J. P., Ribera d’ Alcalà, M. & Falciatore, A. Light sensing and responses in marine microalgae. *Curr. Opin. Plant Biol.* **37**, 70–77 (2017). 28456112 10.1016/j.pbi.2017.03.005

[24] Falciatore, A., D’Alcalà, M. R., Croot, P. & Bowler, C. Perception of environmental signals by a marine diatom. *Science* **288**, 2363–2366 (2000). 10875921 10.1126/science.288.5475.2363

[25] Duanmu, D., Rockwell, N. C. & Lagarias, J. C. Algal light sensing and photoacclimation in aquatic environments. *Plant Cell Environ.* **40**, 2558–2570 (2017). 28245058 10.1111/pce.12943 PMC5705019

[26] Jaubert, M. et al. Sensing and signalling in diatom responses to abiotic cues. *Mol. Life Diatoms*10.1007/978-3-030-92499-7_21 (2022).

[27] Dumas, L., Chazaux, M., Peltier, G. & Johnson, X. Cytochrome b6f function and localization, phosphorylation state of thylakoid membrane proteins and consequences on cyclic electron flow. *Photosynth. Res.* **129**, 307–320 (2016). 27534565 10.1007/s11120-016-0298-y

[28] Kroth, P. G. et al. A model for carbohydrate metabolism in the diatom Phaeodactylum tricornutum deduced from comparative whole genome analysis. *PLoS One* **3**, e1426 (2008). 18183306 10.1371/journal.pone.0001426 PMC2173943

[29] Lavaud, J. & Kroth, P. G. In diatoms, the transthylakoid proton gradient regulates the photoprotective non-photochemical fluorescence quenching beyond its control on the xanthophyll cycle. *Plant Cell Physiol.* **47**, 1010–1016 (2006). 16699176 10.1093/pcp/pcj058

[30] Marques da Silva, J., Duarte, B. & Utkin, A. B. Travelling expenses: The energy cost of diel vertical migrations of epipelic microphytobenthos. *Front. Mar. Sci.* **7**, 1–10 (2020).32802822

[31] Moerdijk-Poortvliet, T. C. W. et al. Seasonal changes in the biochemical fate of carbon fixed by benthic diatoms in intertidal sediments. *Limnol. Oceanogr.* **63**, 550–569 (2018).

[32] Coelho, H., Vieira, S. & Serôdio, J. Endogenous versus environmental control of vertical migration by intertidal benthic microalgae. *Eur. J. Phycol.* **46**, 271–281 (2011).

[33] Happey-Wood, C. M. & Jones, P. Rhythms of vertical migration and motility in intertidal benthic diatoms with particular reference to pleurosigma angulatum. *Diatom Res.* **3**, 83–93 (1988).

[34] Goss, R. & Lepetit, B. Biodiversity of NPQ. *J. Plant Physiol.* **172**, 13–32 (2015). 24854581 10.1016/j.jplph.2014.03.004

[35] Blommaert, L., Chafai, L. & Bailleul, B. The fine-tuning of NPQ in diatoms relies on the regulation of both xanthophyll cycle enzymes. *Sci. Rep.* **11**, 1–16 (2021). 34140542 10.1038/s41598-021-91483-x PMC8211711

[36] Croteau, D., Jaubert, M., Falciatore, A. & Bailleul, B. Pennate diatoms make non-photochemical quenching as simple as possible but not simpler. *Nat. Commun.*10.1038/s41467-025-57298-4 (2025). 40064865 10.1038/s41467-025-57298-4 PMC11894083

[37] Jakob, T., Goss, R. & Wilhelm, C. Unusual pH-dependence of diadinoxanthin de-epoxidase activation causes chlororespiratory induced accumulation of diatoxanthin in the diatom Phaeodactylum tricornutum. *J. Plant Physiol.* **158**, 383–390 (2001).

[38] Goss, R. & Jakob, T. Regulation and function of xanthophyll cycle-dependent photoprotection in algae. *Photosynth. Res.* **106**, 103–122 (2010). 20224940 10.1007/s11120-010-9536-x

[39] Lavaud, J., Rousseau, B. & Etienne, A.-L. General Features of Photoprotection By Energy Dissipation in Planktonic Diatoms (Bacillariophyceae)1. *J. Phycol.* **40**, 130–137 (2004).

[40] Barnett, A. et al. Growth form defines physiological photoprotective capacity in intertidal benthic diatoms. *ISME J.* **9**, 32–45 (2015). 25003964 10.1038/ismej.2014.105 PMC4274417

[41] Blommaert, L., Huysman, M. J. J., Vyverman, W., Lavaud, J. & Sabbe, K. Contrasting NPQ dynamics and xanthophyll cycling in a motile and a non-motile intertidal benthic diatom. *Limnol. Oceanogr.* **62**, 1466–1479 (2017).

[42] Bailleul, B. et al. Energetic coupling between plastids and mitochondria drives CO_2_ assimilation in diatoms. *Nature* **524**, 366–369 (2015). 26168400 10.1038/nature14599

[43] Croteau, D. et al. Contrasting nonphotochemical quenching patterns under high light and darkness aligns with light niche occupancy in Arctic diatoms. *Limnol. Oceanogr.*10.1002/lno.11587 (2021).

[44] Ruban, A. V., Johnson, M. P. & Duffy, C. D. P. The photoprotective molecular switch in the photosystem II antenna. *Biochim. Biophys. Acta Bioenerg.* **1817**, 167–181 (2012). 10.1016/j.bbabio.2011.04.007 21569757

[45] Morelle, J. et al. The photoprotective behavior of a motile benthic diatom as elucidated from the interplay between cell motility and physiological responses to a light microgradient using a novel experimental setup. *Microb. Ecol.* **87**, 40 (2024). 38351424 10.1007/s00248-024-02354-7 PMC10864569

[46] Blanchard, G. F., Guarini, J. M., Dang, C. & Richard, P. Characterizing and quantifying photoinhibition in intertidal microphytobenthos. *J. Phycol.* **40**, 692–696 (2004).

[47] Casey, J. R., Grinstein, S. & Orlowski, J. Sensors and regulators of intracellular pH. *Nat. Rev. Mol. Cell Biol.* **11**, 50–61 (2010). 19997129 10.1038/nrm2820

[48] Pivato, M. & Ballottari, M. Chlamydomonas reinhardtii cellular compartments and their contribution to intracellular calcium signalling. *J. Exp. Bot.* **72**, 5312–5335 (2021). 34077536 10.1093/jxb/erab212 PMC8318260

[49] Helliwell, K. E. et al. A novel Ca^2+^ signaling pathway coordinates environmental phosphorus sensing and nitrogen metabolism in marine diatoms. *Curr. Biol.* **31**, 978-989.e4 (2021). 33373640 10.1016/j.cub.2020.11.073

[50] Helliwell, K. E. et al. Spatiotemporal patterns of intracellular Ca^2+^ signalling govern hypo-osmotic stress resilience in marine diatoms. *New Phytol.* **230**, 155–170 (2021). 33486789 10.1111/nph.17162

[51] Kleiner, F. H. et al. Cold-induced [Ca^2+^] cyt elevations function to support osmoregulation in marine diatoms. *Plant Physiol.* **190**, 1384–1399 (2022). 35894667 10.1093/plphys/kiac324 PMC9516774

[52] Cooksey, B. & Cooksey, K. E. Calcium is necessary for motility in the diatom amphora coffeaeformis. *Plant Physiol.* **65**, 129–131 (1980). 16661126 10.1104/pp.65.1.129 PMC440280

[53] Cooksey, K. E. Requirement for calcium in adhesion of a fouling diatom to glass. *Appl. Environ. Microbiol.* **41**, 1378–1382 (1981). 16345791 10.1128/aem.41.6.1378-1382.1981 PMC243926

[54] Edgar, L. A. & Zavortink, M. The mechanism of diatom locomotion. II. Identification of actin. *Proc. R. Soc. London. Ser. B. Biol. Sci.* **218**, 345–348 (1983).

[55] Poulsen, N. C., Spector, I., Spurck, T. P., Schultz, T. F. & Wetherbee, R. Diatom gliding is the result of an actin-myosin motility system. *Cell Motil. Cytoskelet.* **44**, 23–33 (1999). 10.1002/(SICI)1097-0169(199909)44:1<23::AID-CM2>3.0.CO;2-D 10470016

[56] McLachlan, D. H., Underwood, G. J. C., Taylor, A. R. & Brownlee, C. Calcium release from intracellular stores is necessary for the photophobic response the benthic diatom navicula perminuta (bacillariophyceae). *J. Phycol.* **48**, 675–681 (2012). 27011084 10.1111/j.1529-8817.2012.01158.x

[57] Helliwell, K. E. et al. Alternative mechanisms for fast Na^+^/Ca^2+^ Signaling in eukaryotes via a novel class of single-domain voltage-gated channels. *Curr. Biol.* **29**, 1503-1511.e6 (2019). 31006567 10.1016/j.cub.2019.03.041 PMC6509283

[58] Murphy, E. A., Kleiner, F. H., Helliwell, K. E. & Wheeler, G. L. Channels of evolution: Unveiling evolutionary patterns in diatom Ca^2+^ signalling. *Plants* **13**, 1207 (2024). 38732422 10.3390/plants13091207 PMC11085791

[59] Flori, S. et al. Diatoms exhibit dynamic chloroplast calcium signals in response to high light and oxidative stress. *Plant Physiol.*10.1093/plphys/kiae591 (2024). 39515781 10.1093/plphys/kiae591 PMC11663583

[60] Wilde, A. & Mullineaux, C. W. Light-controlled motility in prokaryotes and the problem of directional light perception. *FEMS Microbiol. Rev.* **41**, 900–922 (2017). 29077840 10.1093/femsre/fux045 PMC5812497

[61] Serôdio, J., Schmidt, W., Frommlet, J. C., Christa, G. & Nitschke, M. R. An LED-based multi-actinic illumination system for the high throughput study of photosynthetic light responses. *PeerJ* **6**, 5589 (2018). 10.7717/peerj.5589 PMC6128260 30202661

[62] Serôdio, J. et al. Efficiency of photoprotection in microphytobenthos: Role of vertical migration and the xanthophyll cycle against photoinhibition. *Aquat. Microb. Ecol.* **67**, 161–175 (2012).

[63] Reed, P. [56] Ionophores. In *Methods in Enzymology* (ed. Reed, P.) (Academic Press, 1979). 10.1016/0076-6879(79)55058-7 379502

[64] Xie, X., Gao, S., Gu, W., Pan, G. & Wang, G. Desiccation induces accumulations of antheraxanthin and zeaxanthin in intertidal macro-alga ulva pertusa (Chlorophyta). *PLoS One* **8**, 1–6 (2013). 10.1371/journal.pone.0072929 PMC3764160 24039824

[65] Casper-lindley, C. & Björkman, O. Nigericin insensitive post-illumination reduction in fluorescence yield in Dunaliella tertiolecta (chlorophyte). *Photosynth. Res.* **50**, 209–222 (1996). 24271960 10.1007/BF00033120

[66] Frankenbach, S., Schmidt, W., Frommlet, J. C. & Serôdio, J. Photoinactivation, repair and the motility−physiology trade-off in microphytobenthos. *Mar. Ecol. Prog. Ser.* **601**, 41–57 (2018).

[67] Serôdio, J., Ezequiel, J., Frommlet, J., Laviale, M. & Lavaud, J. A method for the rapid generation of nonsequential light-response curves of chlorophyll fluorescence. *Plant Physiol.* **163**, 1089–1102 (2013). 24067245 10.1104/pp.113.225243 PMC3813635

[68] Alkimin, G. D. et al. Science of the total environment evaluation of pharmaceutical toxic effects of non-standard endpoints on the macrophyte species lemna minor and lemna gibba. *Sci. Total Environ.* **657**, 926–937 (2019). 30677958 10.1016/j.scitotenv.2018.12.002

[69] Genty, B., Briantais, J. M. & Baker, N. R. The relationship between the quantum yield of photosynthetic electron transport and quenching of chlorophyll fluorescence. *Biochim. Biophys. Acta Gen. Subj.* **990**, 87–92 (1989).

[70] Klughammer, C. & Schreiber, U. Complementary PS II quantum yields calculated from simple fluorescence parameters measured by PAM fluorometry and the saturation pulse method. *PAM Appl. Notes* **1**, 27–35 (2008).

